
JOURNAL INFORMATION
==============================
NLM Title Abbreviation: ScientificWorldJournal
Journal ID: ScientificWorldJournal
NLM Journal ID: 101131163
Journal ID: TSWJ

The Scientific World Journal

ISSN: 2356-6140
EISSN: 1537-744X
Publisher: Wiley

ARTICLE INFORMATION
==============================
PMCID: PMC5917183
PMID: 17013528
DOI: 10.1100/tsw.2006.203
Article ID: 805647
Article version: 1
Subjects: Editorial

Evaluating Side Effects of Human Exposure to Noxious Agents

Matthopoulos Demetrios P. *
Department of Environmental and Natural Resources Management, Ioannina University Agrinio Campus, 30100 Agrinio, Greece
*Demetrios P. Matthopoulos: dmatthop@cc.uoi.grAcademic Editor: Demetrios Matthopoulos

Publication date: 2006
Electronic publication date: 2006 Sep 25
Volume: 6
First page: 1238
Last page: 1240
Received 2006 Aug 16; Accepted 2006 Aug 16
Copyright: Copyright © 2006 Demetrios P. Matthopoulos.
Copyright year: 2006
License: This is an open access article distributed under the Creative Commons Attribution License, which permits unrestricted use, distribution, and reproduction in any medium, provided the original work is properly cited.
License URL: https://creativecommons.org/licenses/by/3.0/

==============================
Editorial
Special Issue: Role of Microscopy on Environmental Factors Influencing Human Genetic Material
*TheScientificWorldJOURNAL* (2006) 6, 1238–1240
ISSN 1537-744X; DOI 10.1100/tsw.2006.203

TheScientificWorldJOURNAL
www.thescientificworld.com

### Evaluating Side Effects of Human Exposure to Noxious Agents

Demetrios P. Matthopoulos

*Department of Environmental and Natural Resources Management, Ioannina University, Agrinio Campus, Seferi 2, 30100 Agrinio, Greece*

**E-mail**: dmatthop@cc.uoi.gr

*Received August 16, 2006; Accepted August 16, 2006; Published September 25, 2006*

---

The anthropogenic environment as developed during the last 2 centuries rendered humanity vulnerable to a large number of environmentally noxious agents to which it had never been exposed. These agents, from their initial introduction into the ecosystem, directly or indirectly, continuously exert their effect on both living and nonliving systems. The result of this exposure will be evaluated by future generations.

Over their evolutionary process, living systems have developed mechanisms to encounter naturally existing noxious conditions. These mechanisms gradually were developed in a way to protect life and assure its future evolution. Technological development over the last 2 centuries posed a serious threat to the evolutionary process of all living systems by introducing new and unknown noxious agents. These agents exert their effect either on biochemical pathways or on the DNA content of organisms. Regardless their target in living systems, they can be toxic, mutagenic, or carcinogenic.

Humans are exposed to these agents either consciously or unconsciously. Conscious exposure occurs either occupationally or in lifestyle and can be controlled, while unconscious exposure is very dangerous.

Electromagnetic radiation as well as chemicals used in industry, agriculture, services, food processing, or even for research and medical practice are among noxious agents introduced by humans. Human activities determine the rate by which these agents are introduced into the global ecosystem. Some of them have a short half-life, being naturally degraded in the atmosphere, ground, or water, while others are reasonably stable, exerting their side effects over long periods and areas. In addition, a considerable number of these agents exert their undesirable effect as products per se, while some of their degradation products are not considered noxious. However, a number of such agents are naturally degraded into byproducts that are more or less dangerous. Such byproducts sometimes are stable over long periods, can be accumulated in the ground or pass through it to the underground water pools, transmitting their action over long distances and periods.

The present special issue is devoted to the various cytogenetic tests by which scientists try to elucidate the problem of human exposure to such noxious agents, as they confront them, consciously or unconsciously, in daily life. Conventional and sophisticated techniques are used nowadays. The cytogenetic techniques of choice for side effect evaluation are the micronucleus test (MN), the comet assay or single cell gel electrophoresis (CA or SCGE) test, the sister chromatid exchange (SCE), and the premature chromosome condensation (PCC) technique. In the meantime, combinations of various microscopic and molecular techniques are used for the study of the molecular events that result in the induced DNA damage from such agents.

The micronucleus test, initially introduced by Heddle[1] for the study of chromosome damage, became an established technique for the evaluation of noxious agents side effects by Fenech and Morley[2]. Noxious agents exert either aneugenic or clastogenic effects. In aneugenic action, they

©2006 with author.
Published by TheScientificWorld, Ltd.; www.thescientificworld.com interfere with the mitotic apparatus and so chromosomes are disoriented and result in the cytoplasm instead of being incorporated in the daughter nuclei during cytokinesis. In clastogenic action, they interfere with DNA, causing brakes that result in acentric chromosomal fragments that are disoriented during mitosis. Thus, disoriented chromosomal material is being organized in the cytoplasm as micronuclei. The micronucleus test can either be used *in vivo* to evaluate the damage induced after human exposure to noxious agents or *in vitro* to evaluate either the potency of existing and new chemicals or the side effects of other environmentally noxious agents. The results of this technique were initially estimated by microscopic observations, while nowadays flow cytometry is being used because it is faster and statistically more reliable due to the large number of cells analyzed[3].

The comet assay or the single cell electrophoresis test introduced by Singh et al.[4] provides information on the extent of the induced damage. It is widely used either for *in vitro* or *in vivo* DNA damage evaluation studies. Its results derive from microscopic observation of the nuclear content after being exposed to constant electric field. Depending on the extent of DNA damage, nuclei remain either intact or appear as comets of variable length.

The evaluation of exchanges between sister chromatids, representing homologous recombination events, initially introduced by Taylor and colleagues[5,6] and adapted by Huang[7], became a technique of choice in the study of side effects of toxicologically harmful agents to humans that have been exposed[8]. The evaluation of the induction of such chromatid exchanges is a means to study the potential of various environmental factors in affecting the genetic material of organisms.

The premature chromosome condensation, which is the induction of cellular DNA to be organized into chromosomes well before mitosis, allows cytogeneticists to discriminate between clastogenic and aneugenic potential of noxious agents. The technique initially introduced by Johnson and Rao[9] was further adapted by Pontecorvo[10] and advanced by Coco-Martin and Begg[11]. Combining premature chromosome condensation with other cytogenetic and molecular techniques, scientists are equipped with a powerful tool to study the genomic changes resulting from the exposure to various environmental agents.

Using the above-referred techniques, we are able to either examine the damage induced by various noxious agents or study their harmful potential.

Based on incoming information, scientists are drawing measures for protecting ourselves and estimating the risk from human past exposure to such chemicals, while they are trying to discover alternative and safer chemicals to replace the ones we use today.

Technological development, despite the danger to which it has periled humans and the other organisms, is devoted to ameliorating daily life by searching for safer systems, increased and safe food provision, and finding ways to remediate natural ecosystems suffering from human greed.

### REFERENCES

1. Heddle, J. (1973) A rapid *in vivo* test for chromosome damage. *Mutat. Res.* **18**, 187–190.
2. Fenech, M. and Morley, A.A. (1985) Measurement of micronuclei in lymphocytes. *Mutat. Res.* **147**, 29–36.
3. Nüsse, M. and Kramer, J. (1984) Flow cytometric analysis of micronuclei found in cells after irradiation. *Cytometry* **5**, 20–25.
4. Singh, N.P., McCoy, M.T., Tice, R.R., and Schneider, E.L. (1988) A simple technique for quantitation of low levels of DNA damage in individual cells. *Exp. Cell Res.* **175**, 184–191.
5. Taylor, J.H. (1958) Sister chromatid exchanges in tritium labelled chromosomes. *Genetics* **43**, 515–529.
6. Taylor, J.H., Woods, P.S., and Hughes, W.L. (1957) The organization and duplication of chromosomes as revealed by autoradiographic studies using tritium-labelled thymidine. *Proc. Natl. Acad. Sci. U. S. A.* **43**, 122–128.
7. Huang, C.C. (1967) Induction of a high incidence of changes to X chromosomes of *Rattus (Mastomys) natalensis* by base analogues, viruses and carcinogens. *Chromosoma* **23**, 162–179.
8. Lambert, B., Lindblad, A., Holmberg, K., and Francesconi, D. (1982) The use of sister chromatid exchange to monitor human populations for exposure to toxicologically harmful agents. In *Sister Chromatid Exchanges.* Wolff, S., Ed. Wiley Interscience, New York. pp. 149–182.
9. Johnson, R.T. and Rao, P.N. (1970) Mammalian cell fusion: induction of premature chromosome condensation in interphase nuclei. *Nature* **226**, 717–722.

10. Pontecorvo, G. (1975) Production of mammalian somatic cell hybrids by means of polyethylene glycol treatment. *Somatic Cell Genet*. **1**, 397–400.
11. Coco-Martin J.M. and Begg, A.C. (1997) Detection of radiation-induced chromosome aberrations using fluorescence in situ hybridization in drug-induced premature chromosome condensations of tumour cell lines with different radiosensitivities. *Int. J. Radiat. Biol*. **71**, 265–273.

**This article should be cited as follows:**

Matthopoulos, D.P. (2006) Evaluating side effects of human exposure to noxious agents. *TheScientificWorldJOURNAL* **6**, 1238–1240. DOI 10.1100/tsw.2006.203.
